# Cross-Domain Text Mining to Predict Adverse Events from Tyrosine Kinase Inhibitors for Chronic Myeloid Leukemia

**DOI:** 10.3390/cancers14194686

**Published:** 2022-09-26

**Authors:** Nidhi Mehra, Armon Varmeziar, Xinyu Chen, Olivia Kronick, Rachel Fisher, Vamsi Kota, Cassie S. Mitchell

**Affiliations:** 1 Laboratory for Pathology Dynamics, Department of Biomedical Engineering, Georgia Institute of Technology, Emory University School of Medicine, Atlanta, GA 30332, USA; 2 Division of Hematology and Oncology, Georgia Cancer Center, Augusta University, Augusta, GA 30912, USA; 3 Center for Machine Learning, Georgia Institute of Technology, Atlanta, GA 30332, USA

**Keywords:** chronic myeloid leukemia, tyrosine kinase inhibitor, BCR ABL, adverse event, side effect, toxicity, heterogeneous information network, machine learning, natural language processing

## Abstract

**Simple Summary:**

Tyrosine kinase inhibitor (TKI) therapy is often taken indefinitely by chronic myeloid leukemia (CML) patients. However, little is known about the long-term or under-reported TKI side effects due to limited longitudinal clinical data on these relatively new drugs. We utilize novel cross-domain text mining and machine learning to comprehensively assess relationships between TKIs and possible adverse events (AEs) across 30+ million PubMed publications. The top 10% of predicted AEs were mapped to ten physiological function-based foci: hematology, glucose, iron, cardiovascular, thyroid, inflammation, kidney, gastrointestinal, neuromuscular, and others. Study results provided new insight into the breadth of adverse events with TKI usage. Results-based recommendations were devised for proactive patient monitoring protocols as a function of perceived AE risk. Additionally, AEs were mapped to specific TKI types to assist in personalized TKI selection.

**Abstract:**

Tyrosine kinase inhibitors (TKIs) are prescribed for chronic myeloid leukemia (CML) and some other cancers. The objective was to predict and rank TKI-related adverse events (AEs), including under-reported or preclinical AEs, using novel text mining. First, k-means clustering of 2575 clinical CML TKI abstracts separated TKIs by significant (*p* < 0.05) AE type: gastrointestinal (bosutinib); edema (imatinib); pulmonary (dasatinib); diabetes (nilotinib); cardiovascular (ponatinib). Next, we propose a novel cross-domain text mining method utilizing a knowledge graph, link prediction, and hub node network analysis to predict new relationships. Cross-domain text mining of 30+ million articles via SemNet predicted and ranked known and novel TKI AEs. Three physiology-based tiers were formed using unsupervised rank aggregation feature importance. Tier 1 ranked in the top 1%: hematology (anemia, neutropenia, thrombocytopenia, hypocellular marrow); glucose (diabetes, insulin resistance, metabolic syndrome); iron (deficiency, overload, metabolism), cardiovascular (hypertension, heart failure, vascular dilation); thyroid (hypothyroidism, hyperthyroidism, parathyroid). Tier 2 ranked in the top 5%: inflammation (chronic inflammatory disorder, autoimmune, periodontitis); kidney (glomerulonephritis, glomerulopathy, toxic nephropathy). Tier 3 ranked in the top 10%: gastrointestinal (bowel regulation, hepatitis, pancreatitis); neuromuscular (autonomia, neuropathy, muscle pain); others (secondary cancers, vitamin deficiency, edema). Results suggest proactive TKI patient AE surveillance levels: regular surveillance for tier 1, infrequent surveillance for tier 2, and symptom-based surveillance for tier 3.

## 1. Introduction

Chronic myeloid leukemia (CML) is an uncommon type of cancer in the bone marrow that targets early myeloid cells. It results in an increase in impartially mature cells that offsets normal myeloid cells. Comprising approximately 15% of all leukemias, the majority of CML cases are cytogenetically characterized by the presence of the Philadelphia (Ph+) chromosome [1]. Ph+ results from the fusion of the Abelson (ABL) tyrosine kinase gene of chromosome 9 and the BCR gene at chromosome 22. CML is most frequently diagnosed among adults of age 65–74 years. However, pediatric, adolescent or young adult CML diagnoses are increasing [2]. In 2021, chronic myeloid leukemia had an estimated 9110 new cases and 1220 new deaths [1].

The advent of BCR ABL targeting tyrosine kinase inhibitors (TKI) as a therapy for CML has substantially improved patient outcomes [3,4]. Currently, five TKIs are widely utilized for CML treatment: imatinib, dasatinib, nilotinib, bosutinib, and ponatinib. Nowadays, patients with CML boast a life expectancy similar to the general population. With improved life expectancy, the goals of CML treatment have shifted from short-term disease management towards the long-term quality of life [5]. Namely, treatments aim to achieve deep molecular response, where blood levels of BCR ABL transcript measured via polymerase chain reaction (PCR) are less than or equal to 0.01% on the International Scale (IS), which is equivalent to a 4-log reduction in BCR ABL transcript with ≤1:10,000 of measured transcripts still possessing the mutation [6]. For many patients, obtaining and maintaining a deep molecular response via ongoing TKI therapy makes CML more analogous to a chronic condition. Nonetheless, these powerful TKIs are riddled with adverse effects.

More recently, the possibility of treatment-free remission (TFR) has been of great interest. TFR is where patients with a deep molecular response cease to continue TKI therapy if they meet specific criteria. In the United States, the most successful guidelines for TFR patient selection include those CML patients who meet all the following criteria: patients who did not have a high Sokal score at initial diagnosis, never progressed past chronic phase, did not possess TKI resistant mutations, have been on TKI therapy for 8 years, and have had a deep molecular response for >2 years at the time of TFR trial initiation [7]. Several trials have commenced to assess relapse rates in patients who undergo TFR, including but not limited to the French STIM trial [8], the TWISTER study in Australia [9], the EURO-SKI study [10], the ENESTfreedom study [11], ENESTop study [12], and the DASFREE study [13]. Unfortunately, many patients who attempt TFR relapse are unsuccessful, with the majority relapsing within 6 months. Between 40–60% of patients ultimately relapse and must resume TKI therapy [14]. Based on current TKI therapies and corresponding clinical evidence, the majority of CML patients require TKI therapy indefinitely (i.e., for the remainder of their natural life).

While TKIs have been very effective, these powerful drugs are not benign. Due to the ubiquitous nature of the drugs’ targets in the body, there is a litany of adverse events (AEs) and side effects associated with TKI usage [15]. Studies have shown cardiovascular, musculoskeletal, and gastrointestinal disorders to all be associated with TKI therapy [13,16,17]. Moreover, TKIs are not considered safe to use during pregnancy [18]. Poor quality of life due to adverse events or side effects can result in decreased patient TKI compliance, which may lead to drug resistance [19,20,21]. Thus, a comprehensive understanding of AEs or side effects is critical for patient management, including patient monitoring, selection of the optimal TKI given the patient’s medical history, and for the developing future, more targeted TKI therapies that may reduce undesired effects [16].

Most TKI therapies are relatively new, and CML is a relatively uncommon leukemia. Thus, there is a limited pool of clinical cohort studies on less common AEs or preclinical AEs that may otherwise go undetected. Here, we propose cross-domain text mining as an innovative opportunity to circumvent the challenges associated with the lack of large-scale longitudinal clinical cohort studies across under-explored physiology. The goal is to utilize integrated text-mined relationships across seemingly disparate domains (oncology, cardiology, neurology, etc.) to predict new or previously undetected/under-reported AEs. Cross-domain literature mining leverages a heterogeneous information network or “knowledge graph”. The graph connects multifactorial and multi-scalar relationships to genes, proteins, processes, disease or syndromes, drugs, etc. A knowledge graph uses extracted text relationships to create a flowchart of connections. A scientist or clinician can typically deduce relationships that are a couple of connections away from the target node (e.g., disease or drug) interest. However, more distant cross-domain relationships further from the target are difficult to manually deduce. Cross-domain text mining is performed with knowledge graph-based text mining software, such as SemNet, to extract relationships from millions of indexed abstracts in the PubMed database [22,23].

Here, we present a novel cross-domain text mining method with link prediction and hub node network analysis to predict and rank lesser-known AEs relevant to tyrosine kinase regulation. The idea for hub node network analysis was borrowed from bioinformatics where network analysis is used to trace downstream gene relationships to “hub” genes [24,25,26]. Analysis of knowledge graphs and prediction of novel links is usually limited by the path link to the target of interest. However, hub node network analysis extends the ability to identify or predict more distant or less obvious links. Hub node network analysis examines neighboring relationships around connected “hub nodes” and not simply the target of interest. Therefore, links to TKI-related AEs can be efficiently predicted that may otherwise be missed.

In summary, the objective of this study was to perform an in-depth assessment of TKI AEs. Standard bag-of-words text clustering of the CML-specific literature was performed to map the most common AEs to specific TKI drugs. However, novel cross-domain text mining with link prediction and hub node network analysis identified and ranked less obvious or under-reported AEs linked to physiological changes in tyrosine kinase. The relevance of this study’s results is: (1) improved awareness of TKI AEs for surveillance, detection, and management; (2) improved personalized TKI therapy selection based on patient history and specific TKI AE profile; (3) aided design of future TKIs to attenuate or eliminate AEs.

## 2. Materials and Methods

The overall goal was to identify and rank potential adverse events (AEs) or side effects due to TKI therapy in CML patients using innovative text mining techniques. Methods consisted of two major analyses: (1) Bag-of-words cluster analysis to connect AEs to specific TKI drug types based on text mining of CML-specific literature; (2) novel cross-domain text mining across all 30+ million biomedical articles in PubMed to identify and rank lesser-known possible AEs linked to tyrosine kinase.

### 2.1. Cluster Analysis of TKI-Specific Adverse Events

First, peer-reviewed CML journal articles in PubMed were extracted to assess the statistical associations between highly occurring AEs and specific TKI drugs (imatinib, nilotinib, dasatinib, bosutinib, ponatinib) based on the presence of specific terms in a document (a specific journal article) and the overall corpus (all included CML articles). After conducting PubMed search queries, 2575 abstracts were retrieved through the year 2021. An example query was “CML AND side effects OR adverse events”. All queries included synonyms to look for any associated condition, comorbidity, or toxicity. The abstracts were parsed and common stop words (“a”, “an”, “the”, “but”, etc.) and punctuation were removed. Abstracts were then tokenized, lemmatized, and cleaned in Python using the Natural Language Toolkit library. A bag-of-words technique [27] was used to vectorize abstracts and transform them into a term frequency-inverse document frequency (TF-IDF) matrix. TF-IDF calculates how relevant a word is in a corpus. The meaning of TF-IDF increases proportionally to the number of times in the text a word appears but is compensated by the word frequency in the corpus. The TF-IDF matrix for this study had dimensions of (2575 × 1621). Thus, 1621 terms occurring in between 1% and 85% of the abstracts were included for analysis. For this portion of the analysis, only abstracts were processed to reduce bias from repetitive or less relevant text.

K-means clustering [28] was used to showcase the distinct adverse event and side effect profiles associated with specific TKIs used to treat CML. The TF-IDF matrix was used as the input for k-means clustering, which was implemented using Scikit-learn in Python. The data was reduced to 50 components using TruncatedSVD [29]. Finally, t-Distributed Stochastic Neighbor Embedding (t-SNE) was used to further reduce the dimensionality for 2D visualization [29].

After examining the top 200 terms with the highest term frequency inverse document frequency (TF-IDF) scores in each cluster, clusters 4, 7, 8, 10, and 16 were identified to be relevant to specific TKIs and their associated undesired effects. A list of the top terms related to AEs and toxicity was identified from inspecting the aforementioned terms in each of the five clusters. The highest frequency of occurrence for each term in the clusters was noted to identify specific AE profiles associated with each of the TKIs. For each of these clusters, fold differences were calculated for each term to compare its frequency within the cluster to its frequency within the corpus of abstracts. Pairwise Chi-Square tests were used to determine whether each of the five clusters identified was significantly different from each other. A family-wise alpha of 0.05 was utilized with a Bonferroni correction for multiple comparisons to minimize Type I error.

### 2.2. Overview of Cross-Domain Text Mining of Conditions Linked to Tyrosine Kinase

The TKI-specific clusters from the bag-of-word analysis of peer-reviewed CML journal article abstracts are helpful for assessing known drug-specific TKI AEs. However, a different approach is needed to assess potential AEs not yet measured or mentioned in CML literature. One way to assess distant, interconnected relationships across domains is to utilize a knowledge graph. Machine learning algorithms and software, such as SemNet, can be used to rank relationships within a large knowledge graph to one or more targets of interest. This study used the original SemNet [23] software for this purpose given work was completed before the final release of SemNet 2.0 [22]. Note the primary difference between the versions is that SemNet 2.0 has a much faster compute time.

**Figure 1:**
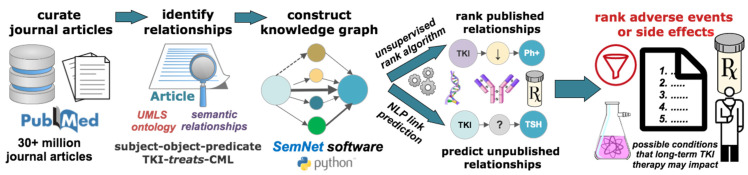
Overview of cross-domain text mining relationships extracted from peer-reviewed articles in PubMed and connected into a knowledge graph. Subject-object-predicate triples are extracted from the text of journal articles, which relate two or more nodes or “concepts” of interest. The knowledge graph ontology is specified by the Unified Medical Language System (UMLS). Example node types include pharmacological substance, gene or genome, neoplastic process, organic chemical, cell component and amino acid/protein. Predication types include “interacts”, “associated”, “part of”, “treats”, “inhibits”, “stimulates”, “causes”, and “is a”. The open-source Python-based biomedical literature-based discovery software, SemNet [23], was used to identify rank relationships between possible adverse events or side effects due to tyrosine kinase inhibition. A standard SemNet simulation was used to rank published relationships connecting undesired conditions to tyrosine kinase. A natural language processing (NLP) link prediction algorithm [30] was used in combination with SemNet to identify relationships that are not explicitly in the knowledge graph. Link prediction uses existing published relationships in the knowledge graph to predict novel relationships, which are either unpublished or, minimally, do not exist in the graph. Results are then synthesized and ranked to suggest adverse events or side effects that should be monitored as part of TKI therapy.

Two types of knowledge graph text mining were performed: the standard unsupervised rank aggregation algorithm of SemNet to assess published relationships in the knowledge graph [23] and a link prediction module to assess predicted unpublished relationships [30]. Link prediction has been used by other studies as well to predict novel links of importance from biomedical knowledge graphs [31,32,33]. Hub node network analysis was developed and implemented to extend the coverage of link prediction for this study. Collectively, relationships predicted as important suggest possible conditions that should be monitored during TKI therapy. Figure 1 illustrates the knowledge graph pipeline utilized in this work.

Unlike a clinical meta-analysis, text mining does not select traditional “patient cases”. Rather, text mining traverses a large knowledge graph of interconnected relationships derived from biomedical text to find and rank relationships to a target of interest. An example of an extracted biomedical text relationship is Philadelphia chromosome CAUSES chronic myeloid leukemia. Thus, one of the limitations of cross-domain text mining is that it does not presently allow for the inclusion of specifics such as dose size, disease stage, or time since treatment started. Searching and analyzing the knowledge graph is limited by its organizing ontology. In this study, the biomedical knowledge graph ontology is the Unified Medical Language System (UMLS). On the other hand, the strength of cross-domain text mining is its breadth to integrate massive amounts of relationships across multiple domains. Thus, cross-domain text mining can pinpoint important or novel relationships that might otherwise go undetected in a standard quantitative clinical meta-analysis due to sample size, study inclusion or exclusion criteria, or unintentional human bias. 

### 2.3. Examination of Existing Published Nodes to CML and Tyrosine Kinase with SemNet

**Figure 2:**
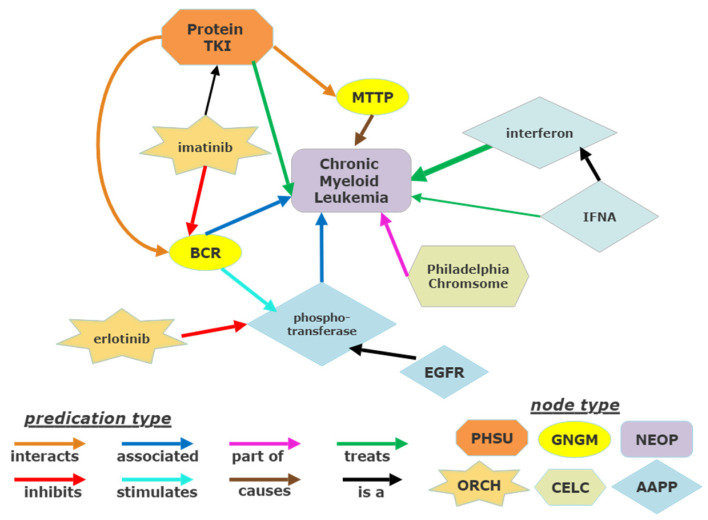
How to read a knowledge graph derived from text-mined biomedical relationships. Text is mined from 30+ million PubMed articles to make a heterogeneous information network, or knowledge graph, of relationships. This example is a highly pruned (>99.9% pruned) subgraph of the larger knowledge graph. The actual knowledge graph is so large and intertwined that it would be completely intractable to the human eye. The shapes are called nodes and represent concepts from biomedical literature. The type of shape represents the ontology of the node, called a node type. Example node types shown include pharmacological substance (PHSU), gene or genome (GNGM), neoplasm (NEOP), organic chemical (ORCH), cell component (CELC) and amino acid, peptide, or protein (AAPP). The ontology for node types and predication types is dictated by the Unified Medical Language System (UMLS). The lines represent predication types, or relationships, between the nodes. The different colors represent a different predication type. The line thickness represents the degree weighted frequency of the relationship in the corpus. Relationships are read in head-PREDICATION-tail format. Examples: imatinib-IS A-protein TKI and imatinib-INHIBITS-BCR.

Examination of existing published nodes relating CML and tyrosine kinase was performed in SemNet. SemNet is a semantic inference network and biomedical concept knowledge graph that uses unsupervised ranking algorithms to reveal hidden connections from PubMed’s nearly 30 million indexed abstracts [22,23]. A user can run a simulation for a particular node of interest, which is specified as the “target node”, for which SemNet will search the knowledge graph and return a list of “source nodes” that are connected to the target node(s). Each connection has both a predication type that represents the association between the two nodes, as well as a magnitude that represents the prevalence of the connection within the PubMed literature. A highly pruned subgraph of the larger knowledge graph utilized is shown as an example in Figure 2. Each node has a particular node type to help the user methodically filter for desired source nodes. For instance, if a user wanted to examine all the published genetic factors involved in chronic myeloid leukemia, they could run a simulation with a target node of “chronic myeloid leukemia” and filter the resulting source nodes for the “gene or genome” (GNGM) node type. Node names and node types follow the Unified Medical Language System (UMLS) ontology.

SemNet simulations were utilized to examine diseases and therapeutic substances that might be associated with TKI therapy for CML. The simulation parameters were set to filter for two particular node types in the knowledge graph: Diseases or Syndromes (DSYN) and Therapeutic or Preventive Procedure (TOPP). These parameters were specified to focus on any immediate diseases or therapeutic substances within one connection of the specified target node: CML. After the simulation was run, a comprehensive list of 308 treatments and diseases related to CML were retrieved. The list was subsequently analyzed to look for existing trends or novel relationships derived from the current CML literature. HeteSim scores were utilized to rank conditions that were of most importance to CML and tyrosine kinase.

### 2.4. Link Prediction with Hub Node Network Analysis to Predict Cross-Domain Relationships

We utilize our previously published link prediction algorithm [30] in conjunction with the newly described hub node network analysis method to predict novel AEs related to tyrosine kinase. These “novel” AEs include conditions that may not be directly connected to the CML node in the knowledge graph.

#### 2.4.1. Motivation for Hub Node Network Analysis Adapted to Knowledge Graphs

Traditional knowledge graph analysis and link prediction identify links directly connected to a target of interest. For example, SemNet used a standard path link of 2 to compute features for relevance rankings. This means that potentially relevant nodes further from the target may be missed. Expanding the path link, alone, is computationally very expensive and exponentially increases unwanted noise in the resultant rankings.

A similar problem has been cited in large bioinformatics gene-gene networks. Bioinformatics network analysis of hub genes effectively enable scientists to tie changes in downstream genes to highly connected gene hubs [24,25,26]. The same idea is applied here to knowledge graphs. Thus, hub node network analysis effectively increases the path link of the knowledge graph analysis in a “smart” manner. The method prioritizes examination of adjacent, highly connected hub node neighborhoods, which are most likely to contain relevant physiological relationships to the target.

#### 2.4.2. Implementation of Hub Node Network Analysis with Link Prediction

**Figure 3:**
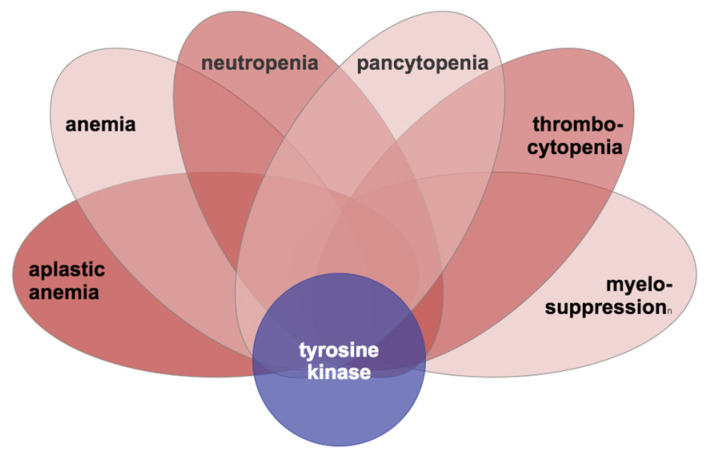
Conceptual overview of hub node network analysis to construct neighborhoods that find more distant or less represented connections to tyrosine kinase in the knowledge graph. The six leaves shown represent the hub nodes (aplastic anemia, anemia, neutropenia, pancytopenia, thrombocytopenia, and myelosuppression) used to generate hub neighborhoods for link prediction. Link prediction and SemNet simulation predicted disease or syndrome (DSYN) nodes related to tyrosine kinase that could be relevant adverse events (AEs) for CML patients on TKI therapy.

Six nodes were specified as “hub nodes” for making the hub node networks: aplastic anemia, anemia, neutropenia, pancytopenia, thrombocytopenia and the more general node, myelosuppression. These nodes were selected as hubs based on their maximal connectivity and their shared overlap between the CML and tyrosine kinase nodes in the initial SemNet simulations described in Section 2.3. A pictorial diagram to illustrate the hub node network analysis concept adapted to natural language processing knowledge graphs is shown in Figure 3.

Once hubs were selected, link prediction was performed using our previously published algorithm [30]. Specifically, entity prediction generated a list of nodes with a specified relationship type to the six node hubs: CAUSES, PRECEDES, AUGMENTS, DISRUPTS, STIMULATES, and AFFECTS. Included UMLS node types were: Amino Acid Peptide or Protein (AAPP), Biologically Active Substance (BACS), Disease or Syndrome (DSYN), Gene or Genome (GNGM), and Pharmacologic Substance (PHSU). The unsupervised graph algorithm, TransE [30] generated lists of nodes predicted to have a relationship with each hub node. A degree normalized weight value represented the predicted strength of the relationship between the head and tail node. The weight values were used to standardize and prune the number of nodes included in the hub node neighborhoods.

The resultant nodes that comprised the hub neighborhoods became SemNet targets for DSYN conditions associated with tyrosine kinase. Pairwise comparisons were performed in SemNet between mutually highly ranked nodes related to tyrosine kinase and the hub neighborhood. To reduce noise, generic nodes such as “protein” were removed. To enable aggregation across separate hub node simulations, the HeteSim scores were recorded and normalized to fall between 0 and 1.

#### 2.4.3. Selection and Validation of Most Relevant Nodes and Aggregation into Tiers and Foci

Approximately the top 10% of normalized, highly ranked nodes aggregated from the hub node simulations were isolated for human-in-the-loop validation. The top 10% were chosen on the basis of the most novelty (using the SemNet novelty score) and overall predicted relevance (using the SemNet HeteSim score). Three tiers were used to classify foci of conditions or AEs predicted to be relevant to TKI therapy for CML. Tier 1 included the top 1% of predicted node aggregates, tier 2 the top 5% of predicted node aggregates, and tier 3 the top 10% of predicted node aggregates. The foci were defined using physiology-based UMLS ontology to codify highly ranked node aggregates representing a similar physiological or pathophysiological process or condition.

We implemented our previous human-in-the-loop protocol [30] to validate predicted cross-domain links. For example, a predicted relationship such as tyrosine kinase-AFFECTS-glucose may have sufficient physiological evidence in diabetes literature but not explicitly in the CML literature. Full-text literature context for predicted relationships was manually reviewed by a team of trained curators. Each predicted relationship was classified as “agrees”, “disagrees”, or “missing”. All AEs included in the tiers and foci passed human-in-the-loop validation with real-world evidence that the relationship was reasonably plausible. Example evidence of the predicted link between tyrosine kinase and the resultant AE conditions are presented in Section 4.

## 3. Results

The primary goal was to identify lesser-known adverse events (AEs) or side effects associated with TKI therapy. A secondary goal was to provide an evidence-based framework to assist clinicians monitoring for AEs and selecting the optimal TKI based on patient history and TKI AE profile.

### 3.1. K-Means Clustering of Adverse Events in the CML TKI Literature

**Figure 4:**
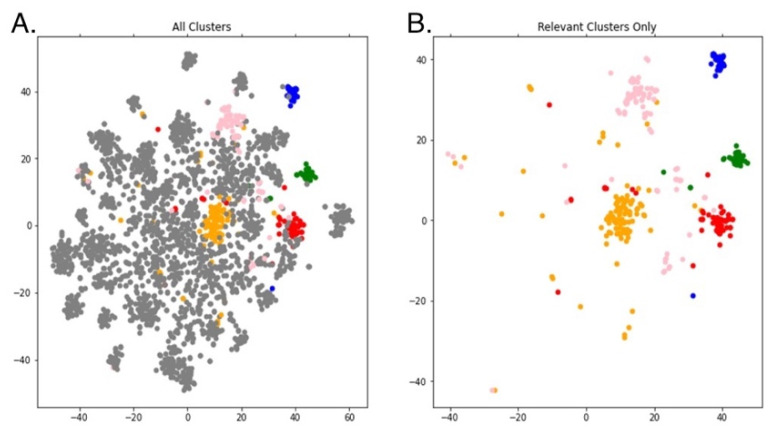
K-means topic clustering of text from downloaded published chronic myeloid leukemia (CML) tyrosine kinase inhibitor (TKI) abstracts. (**A**) Visual representation of the full k-means clustering output. Gray dots represent abstracts unrelated to specific TKIs. The colored dots represent abstracts in the clusters pertaining to five TKIs: bosutinib (blue), imatinib (gold), ponatinib (green), dasatinib (red), and nilotinib (pink). (**B**) Output of the k-means clustering algorithm showing the most relevant abstracts and clusters.

**Figure 5:**
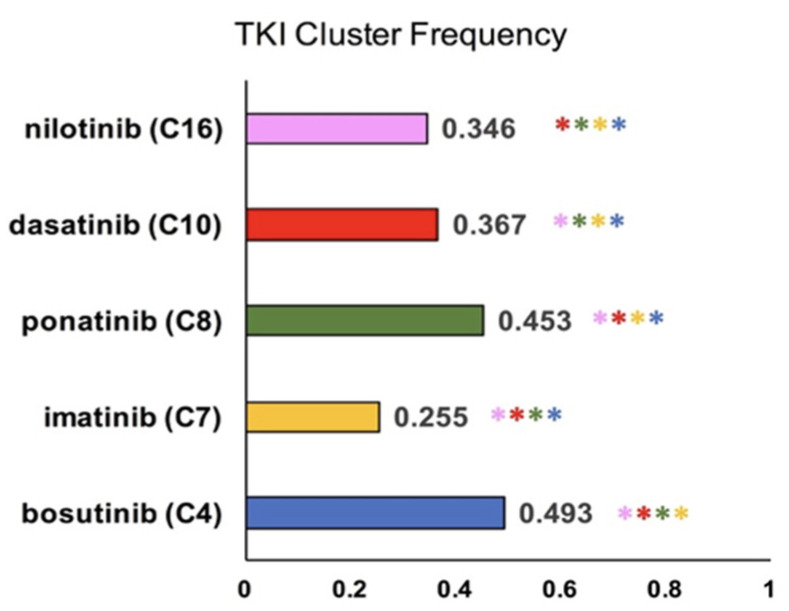
Mapping specific TKIs to AEs. The color of each bar corresponds to the color of the cluster in the previous scatter plot (Figure 4). The frequency of occurrence of each TKI in its related cluster is printed next to each bar. A chi-squared test with Bonferroni correction found a significant difference in fold change. Fold change refers to the difference in frequency of a term within a cluster when compared to its frequency within the corpus as a whole. Color of the asterisk(s) illustrates a significant difference between the cluster depicted in the bar and the cluster depicted by the color of the asterisk. Significance is denoted by family-wise alpha of 0.05.

The intent of k-means clustering of the CML TKI literature was to identify the most common AEs or side effects among specific TKI drugs. Of the thirty clusters generated in Figure 4, five clusters were mapped to a specific TKI based on TKI term frequency within the cluster. Cluster 4 (blue) pertains to bosutinib, cluster 7 (gold) to imatinib, cluster 8 (green) to ponatinib, cluster 10 (red) to dasatinib, and cluster 16 (pink) to nilotinib. Appendix A provides the calculated frequencies used to assign the cluster label for each TKI in Table A1. To statistically analyze the difference between each cluster, pairwise Chi-squared tests were conducted. All five clusters were significantly (*p* < 0.05) different from each other, as depicted in Figure 5.

The frequency of side effects or adverse events within each of the clusters are shown in Table 1. Terms pertaining to gastrointestinal AEs, such as “diarrhea”, “nausea”, and “vomiting” occurred most frequently in cluster 4 (bosutinib). Terms pertaining to hematological and dermatological AEs, such as “edema”, “rash”, “myelosuppression”, “hematological”, and “lesion” occurred most frequently in cluster 7 (imatinib). Additionally, the terms “hepatitis” and “liver” also occurred most frequently in cluster 7 (imatinib). Terms such as “cardiovascular” and “vascular” occurred most frequently in cluster 8 (ponatinib); “platelet” and “arterial” also occurred at high frequencies in cluster 8. Terms such as “pulmonary”, “pleural”, “platelet”, “hypertension”, and “arterial” occurred at the highest frequency in cluster 10 (dasatinib). The only term to occur at the highest frequency in cluster 16 (nilotinib) was “diabetes”; however, terms including “cardiovascular”, “vascular”, and “arterial” also occurred at relatively high frequencies. Finally, terms pertaining to hematological conditions appeared frequently within all five clusters and thus were associated with all TKIs. “Myelosuppression” appeared in all clusters except cluster 16 (nilotinib). The term “hematological” and “platelet” appeared within all clusters except cluster 4 (bosutinib). “Thrombocytopenia” appeared in all clusters.

### 3.2. Initial Cross-Domain Text Mining of Published Relationships to CML

**Figure 6:**
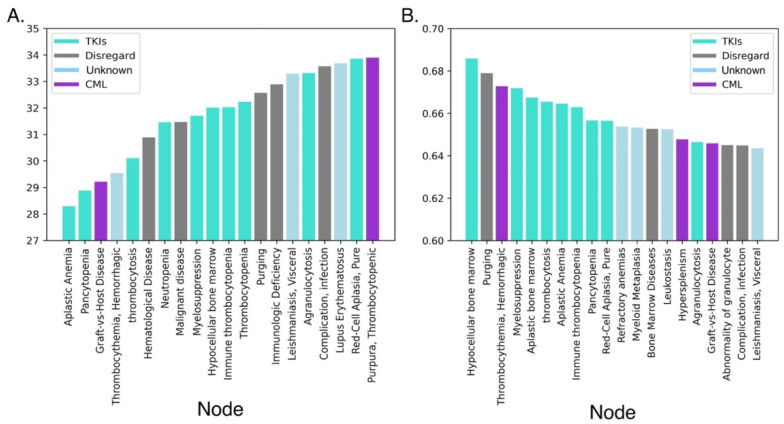
Top twenty Unified Medical Language System (UMLS) nodes related to TKIs and CML. SemNet queried a knowledge graph containing 30+ million journal articles. All nodes shown are of UMLS node type “disease or syndrome” (DSYN). CML and tyrosine kinase were set as SemNet target nodes. Aqua bars correspond to conditions observed in patients on TKIs; purple bars correspond to conditions observed in CML patients on no treatment or on a non-TKI treatment; light blue bars correspond to conditions where no established relationship to CML was found; gray bars correspond to disregarded conditions that were too broad to be informative. (**A**) The top 20 most relevant UMLS node relationships to CML or TKIs based on HeteSim feature relevance. Here, a lower HeteSim score (*y*-axis) correlates to a stronger relationship to the target; thus, from left to right, the strengths of the relationships to the target(s) decrease. (**B**) The top 20 relevant UMLS node relationship to CML or TKIs based on novelty score feature relevance. A higher novelty score correlates to a potentially stronger novel connection (meaning fewer studies have published the relationship, although the algorithm still considers the relationship to be relevant to the target nodes). Novelty decreases from left to right.

Initial SemNet simulations were conducted to assess which conditions were most associated with CML and TKI therapy in a knowledge graph composed of nearly 30+ million PubMed abstracts. With CML as the target node, and DSYN and TOPP as the UMLS source node types, a list of 308 treatments and diseases related to CML was generated. The two SemNet features that were examined were HeteSim score and novelty score. HeteSim score represents the strength of the relationship to the target. Here, a low HeteSim score indicates a stronger relationship between source and target node. The novelty score indicates how potentially novel a connection may be. The resultant TOPP (Therapeutic or Preventive Procedure) source nodes yielded a list of broad procedures without specific processes or drug names and thus were not of interest. The resultant DSYN (Disease or Syndrome) nodes were sorted by their HeteSim scores and novelty scores and compared. The top 20 nodes by HeteSim score and the top 20 nodes by novelty score are shown in Figure 6. Seven out of the top 10 resulting nodes were hematological conditions when ranked by HeteSim. These nodes were aplastic anemia, pancytopenia, thrombocythemia, thrombocytosis, hematological disease, neutropenia, and myelosuppression. Similarly, 6 out of the top 10 resulting nodes were hematological conditions when ranked by relevant novelty (nodes deemed relevant irrespective of the number of reference publications mapped to the relationship). These nodes were thrombocythemia, myelosuppression, thrombocytosis, aplastic anemia, and pancytopenia. The hematological conditions that were found to overlap in both HeteSim-ranked results and novelty-ranked results were aplastic anemia, pancytopenia, thrombocytopenia, neutropenia, and myelosuppression.

The k-means clustering examined only abstracts that specifically included CML and a TKI drug, whereas SemNet examined the entire knowledge graph of cross-domain relationships from all of PubMed. The advantage of cross-domain text mining was that it identified physiological relationships to tyrosine kinase or tyrosine kinase inhibitors that otherwise go undetected if only the CML literature was examined. This extensive heterogeneous information network enables distant or novel relationships to be identified by leveraging the comprehensive biomedical literature. Yet, hematological conditions remained frequent and highly ranked in relevance in both CML text mining and cross-domain text mining. Some of these conditions shown in Figure 6 are related to the presentation of CML, itself (shown in the purple bar); some are specific to CML patients taking TKIs (aqua); and for some conditions, it is not clear to what/where the relationship can be explicitly attributed (light blue). The gray bars in Figure 6 represent known artifacts in SemNet due to the frequency of highly occurring general nodes in the UMLS, such as “complication, infection”. These general nodes were disregarded because they did not add value to the present analysis.

### 3.3. Prediction of Distant or Novel Predicted Connections to Tyrosine Kinase

Initial SemNet simulations examined relationships tied to the target node, CML. However, the inclusion of CML as a target constrained the identification of conditions related to tyrosine kinase pathophysiology that may not have yet been published in the CML literature or where frequency counts are under-represented. In order to examine deeper relationships of potential importance, link prediction [30] and hub node network analysis were utilized. Node neighborhoods were created using six hub nodes identified from the initial CML simulation results: aplastic anemia, anemia, neutropenia, pancytopenia, thrombocytopenia, and myelosuppression. These hub nodes essentially extend the search area to allow for the examination of more distant connections in the knowledge that might be missed when only using “CML” as a target node. Analysis of mutually highly ranked nodes across the hub node neighborhoods predicted relevant relationships to tyrosine kinase that are not necessarily directly connected to CML in the knowledge graph. As such, these results represent new predictions of relevant pathophysiology that may be impacted by tyrosine kinase.

The SemNet feature scores for link prediction with hub node network analysis HeteSim scores were normalized to fall between 0 and 1 to enable uniform comparison and aggregation. As such, the mean occurrence rate and normalized HeteSim scores were aggregated to predict which conditions were most likely to be relevant to TKI therapy. Foci were determined based on three tiers of aggregated cross-domain text mining rankings of conditions predicted as most relevant to tyrosine kinase: tier 1 approximated the top 1% of predicted conditions, tier 2 approximated the top 5% of predicted conditions, and tier 3 approximated the top 10% of predicted conditions. Foci names were UMLS-inspired functional classifications that best represented the associative physiology of the nodes within the foci.

**Figure 7:**
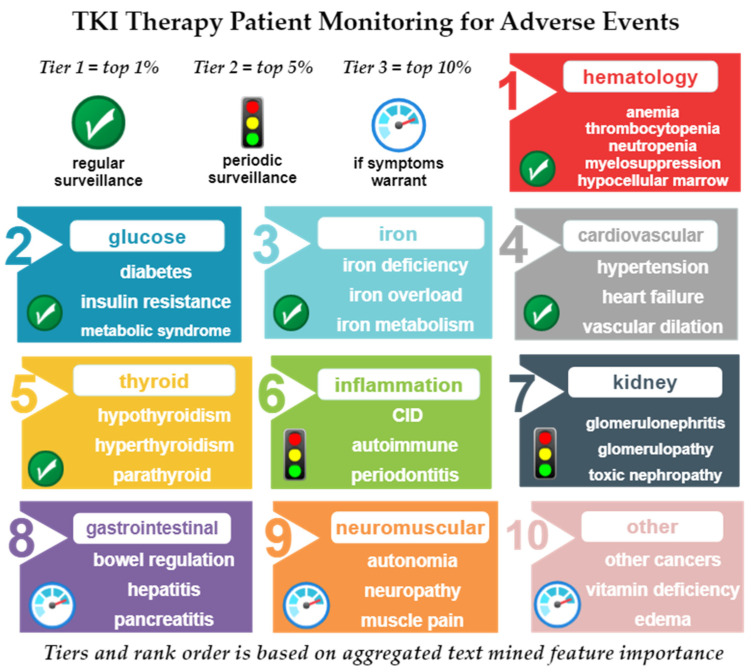
Summary of conditions predicted to be most relevant to tyrosine kinase. Ranked predictions are based on cross-domain literature-based discovery with link prediction and hub node network analysis. Rankings are based on normalized and aggregated HeteSim feature importance scores generated from SemNet. Tier 1 comprised approximately the top 1% of aggregated nodes: hematological conditions (1), glucose (2), iron homeostasis (3), cardiovascular disorders (4), and thyroid disorders (5). The second tier comprised approximately the top 5% of aggregated nodes: inflammation (6) and kidney disorders (7). The third tier comprised approximately the top 10% of aggregated nodes: gastrointestinal symptoms or disorders (8), neuromuscular disorders (9) and other (10). “Other” was used to denote conditions that ranked within the top 10% of aggregated and normalized related nodes but could not be described by a single cohesive functional category. The suggested surveillance categories were based on feature importance by tier: tier 1 suggests regularly scheduled surveillance, tier 2 suggests less frequent (periodic) surveillance, and tier 3 suggests surveillance only when symptoms are present.

Tier 1 included the top 1% of conditions predicted as most relevant to the tyrosine kinase node: hematology condition (1), glucose regulation (2), iron homeostasis (3), cardiovascular conditions (4), and thyroid conditions (5). Tier 2 included the top 5% of conditions predicted as most relevant to the tyrosine kinase node: inflammation (6) and kidney disorders (7). Tier 3 included the top 10% of conditions predicted as most relevant to the tyrosine kinase node: gastrointestinal disorders (8) neuromuscular disorders (9), and other (10). “Other” denoted conditions could not be reasonably described by a single, cohesive physiological category (or foci). Figure 7 illustrates the cross-domain text mining results for the top predicted conditions linked to tyrosine kinase. For each focus, the three highest ranked conditions are listed.

Hematology, cardiovascular, and gastrointestinal conditions were expected to be present based on the results of k-means clustering of frequent terms in the CML-specific literature. However, some of the other conditions identified as relevant to tyrosine kinase in the cross-domain text mining were less obvious. For example, diabetes and kidney disorders only appeared in a single k-means CML literature cluster (cluster 16, nilotinib). Thyroid disorders, iron homeostasis, and inflammation were not among the most frequent terms in the k-means clustering of CML-specific literature.

The three tiers reflect both the prevalence of conditions in the text mined foci, as well as their predicted HeteSim feature relevance to tyrosine kinase. As such, the assigned tier was used to suggest the level of clinical surveillance for CML patients undergoing TKI therapy. Surveillance entails testing for the presence and degree of clinical or subclinical conditions associated with or possibly worsened by TKI therapy. Tier 1 foci are predicted in the present analysis to be prevalent and relevant enough that regularly scheduled surveillance is warranted regardless of patient history. Tier 2 foci are predicted to need periodic surveillance; periodicity is expected to be infrequent, unless a patient has relevant antecedent disease or comorbidities that increase susceptibility to tier 2 conditions. Tier 3 suggests surveillance only when overt or persistent symptoms are present. As such, tier 3 surveillance would be similar to the age-matched general population of non-CML patients.

## 4. Discussion

Specific literature evidence is presented below to provide context and support for CML-specific TKI relationships and cross-domain text mining link predictions between tyrosine kinase and other diseases or syndromes (DSYN). Recall that conditions ranked by cross-domain text mining as “most important” to tyrosine kinase were determined using the normalized HeteSim relevance feature scores to the tyrosine kinase node and connected node neighborhoods. The highly ranked conditions are discussed in the context of possible adverse events or conditions due to tyrosine kinase inhibitor (TKI) therapy. However, as with all relationship-based statistical models, the predictions of cross-domain text mining are associative and not causal. As such, highly ranked conditions related to tyrosine kinase cannot explicitly be interpreted as adverse events caused by tyrosine kinase therapy. Nonetheless, the predictions of associated conditions with tyrosine kinase provide insight for current TKI patient management and future research.

### 4.1. Predicted Tier 1 Adverse Events Likely Tied to TKI Therapy

As noted in Figure 7, the tier one foci comprised the top 1% of predicted nodes relevant to tyrosine kinase. The tier one foci included: 1. hematological conditions; 2. glucose; 3. iron homeostasis; 4. cardiovascular conditions; 5. thyroid disorders. Of these conditions, hematological and cardiovascular disorders were expected based on their term frequency in the k-means clustering in the CML TKI literature (Table 1). However, glucose, iron homeostasis, and thyroid disorders are less discussed in CML literature. Thus, in particular, more awareness of these conditions as possible clinical or subclinical AEs is necessary for optimal TKI therapy patient care.

#### 4.1.1. Hematological Conditions as Adverse Events from TKI Therapy

K-means clustering of CML-specific TKI literature and cross-domain text mining with tyrosine kinase (Figure 4 and Figure 7) found myelosuppressive hematological conditions were among those most frequently cited in CML. Hematological adverse events (namely anemia, neutropenia, thrombocytopenia) were found within each TKI-specific cluster (imatinib, dasatinib, bosutinib, nilotinib, ponatinib). This is not unexpected given the mechanism of action of TKIs, which stop proliferation of Philadelphia chromosome-positive white blood cells. Anemia can be worsened or complicated by iron deficiency, which is the number two predicted adverse event. The persistence of a hematological AE or the deficiency of more than one blood cell type could suggest hypocellular bone marrow, which may occur with myelosuppression. Hypocellular bone marrow was one of the nodes that was highly ranked in the hematological conditions foci.

In summary, the mechanism by which TKIs work means that myelosuppression is an expected possibility. Thus, the goal is to adjust the dose of TKI to minimize myelosuppression that causes anemia, neutropenia, or thrombocytopenia. For patients who are more susceptible to myelosuppression due to other comorbidities or antecedent disease, physicians should select a TKI where myelosuppressive AEs are not as pervasive. In this study, nilotinib was predicted to have the fewest hematological AEs. A regularly performed peripheral complete blood count test can identify signs of myelosuppression. Such a CBC every 4–6 months is currently a common CML standard of care practice. When overt symptoms are present, more specific testing may be warranted to determine if hematological events are being worsened by other conditions, comorbidities, or TKI-related AEs.

#### 4.1.2. Glucose-Related Adverse Events from TKI Therapy

Glucose-related conditions were predicted by cross-domain text mining to be the second most relevant AE with TKI therapy. In the case of glucose, the impact appears to depend on habituation to the drug. Early on, patients on TKIs tend to have lower blood sugar levels or A1c. In fact, imatinib was recently examined in a study as a possible therapeutic for Type I diabetes [34]. While the drug lowered blood sugar initially in the first 12 months, the effect did not last when measured through 24 months [34]. Another published review also discussed the use of TKIs for type 2 diabetes treatment [35]. The results of the present study predicted insulin resistance and metabolic syndromes as highly ranked possibilities with TKI therapy. It is possible that patients initially experience lowering of glucose levels and less insulin resistance. However, with long-term TKI therapy, the innate physiology may habituate and overcompensate, resulting in insulin resistance, pre-diabetes, or metabolic disorder. Nilotinib was most associated with diabetes in the presented k-means clustering text mining in Table 1. Many studies have tied nilotinib to diabetes or minimally impaired glucose metabolism [36,37]. In fact, one clinical panel has recommended for nilotinib to not be the primary therapy for CML patients who also have diabetes [38]. Higher glucose is also associated with iron deficiency anemia; as such these two conditions are intertwined. Insulin resistance can also compound cardiovascular co-morbidities or TKI-related AEs. As such, monitoring of peripheral fasting blood glucose and annual hemoglobin A1c [39] testing could be helpful.

#### 4.1.3. Iron Homeostasis Adverse Events with TKI Therapy

The iron foci ranked number 3 in the cross-domain text mining analysis in its relationship importance to tyrosine kinase. Once again, opposing conditions are identified: iron deficiency (too little iron) and iron overload (too much iron). It is possible both are relevant to TKI therapy. However, it is plausible that iron overload is simply a noisy artifact in this analysis due to its strong relationship with blood transfusions. “Blood transfusion” is a UMLS TOPP node that was strongly connected with the hub nodes used to generate the neighborhood rankings for determining the foci.

Iron deficiency can impact the thyroid [40], which is the fifth-ranked foci. A recent study showed that iron chelator, deferasirox, improved imatinib resistance and increased the clearance of BCR ABL cells [41]. Most of the iron connections in the knowledge graph were related to iron deficiency anemia. As noted in Section 4.1.1, anemia is a relatively common hematological AE with TKI therapy and frequently appeared in the CML-specific literature (Table 1). Iron is important for red blood cell function, anemia prevention, maintaining proper thyroid function, and other important physiological regulatory activities. Thus, regular annual monitoring with an iron panel (peripheral blood levels of iron, iron saturation, and ferritin) is reasonable. Minimally, patients with overt anemia should be checked for iron deficiency as per the usual standard of care.

#### 4.1.4. Cardiovascular Adverse Events with TKI Therapy

The cardiovascular foci ranked fourth in the cross-domain text mining analysis. However, cardiovascular event terms were even more frequent in the k-means clustering of CML-specific literature. Hypertension AE prevalence is approximately 10% for those treated with imatinib, dasatinib, bosutinib, nilotinib [42] but is as high as 30% for patients treated with ponatinib [43]. Heart failure, hypertension, Q-T prolongation, and vascular events are the most commonly monitored cardiovascular AEs among CML patients treated with TKIs. Heart failure ranges from <1% for imatinib or dasatinib to as high as 4% for patients on bosutinib or ponatinib [42]. Q-T prolongation and the associated sudden cardiac death in 0.3% of patients on nilotinib prompted the blackbox warning by the United States Food and Drug Administration (FDA); approximately 1% of dasatinib or imatinib patients had clinically significant Q-T prolongation but much less associated sudden cardiac death [42]. Arterial vascular events occur in approximately 2% of patients for imatinib, 5% for dasatinib, and 11% for nilotinib, although the number for nilotinib was based on a relatively smaller study [42]. Notably, ponatinib was most associated with “cardiovascular” and “arterial” in the k-means clustering of CML literature, followed by nilotinib. Many clinicians agree that cardiovascular risk is one of the most important factors for selecting a TKI [44]. Echocardiogram and electrocardiogram testing is currently recommended to monitor for cardiovascular AEs with TKI therapy [42,44]. The results of the present study concur, given cardiovascular events were predicted as a Tier 1 foci. The frequency of this monitoring should be determined based on patient risk and TKI type. For low-risk patients, monitoring every 2 years would likely be sufficient.

#### 4.1.5. Thyroid Disorders as Adverse Events with TKI Therapy

The thyroid focus was ranked number five in the cross-domain text mining analysis. Again, opposing conditions are identified as hypothyroidism (too little thyroid hormone) and hyperthyroidism (too much thyroid hormone) were the top two conditions in the thyroid foci. In the general [non-CML] population, hyperthyroidism is typically attributed to Grave’s disease, an autoimmune disorder. However, neutropenia is also associated with hyperthyroidism. Certain types of TKIs can cause hyperthyroidism due to their mechanism of action in the down-regulation of tyrosine kinase. Hyperthyroidism was initially thought to be the more common AE during early TKI therapy [45]. However, a recent 2020 study found that hypothyroidism is more prominent than hyperthyroidism in CML patients treated with TKIs [46]. Of 326 patients who were on TKI therapy and had a thyroid disorder as an adverse event, 74% had hypothyroidism and 20% had hyperthyroidism [46]. Of the 326 CML TKI therapy patients with a thyroid AE, hypothyroidism accounted for 73% of cases with imatinib, 75% with dasatinib, 48% with nilotinib, and 87% with bosutinib [46]. It is possible that long-term TKI therapy results in physiological habituation that increases the probability of hypothyroidism over hyperthyroidism.

Cross-doming text mining also predicted parathyroid function as relatively important to the tyrosine kinase node. However, according to normalized HeteSim feature importance scores, the predicted tie between tyrosine kinase and the parathyroid was not as strong as the thyroid, itself. Secondary hyperparathyroidism has been associated with bone marrow fibrosis (hypocelluar bone marrow), which can cause pancytopenia, anemia, thrombocytopenia, or neutropenia. Patients with hyperparathyroidism may not exhibit overt symptoms early in the disease course, making monitoring all the more important. Changes in blood calcium (namely hypercalcemia) or phosphatase (namely hypophosphatemia) due to hyperparathyroidism can impact cardiovascular AEs [47]. In one study, up to 30% of patients taking nilotinib have reported hypophosphatemia [48].

Finally, there are key relationships between anemia, iron homeostasis, and thyroid function which complicates this triad of tier 1 foci of TKI-related AEs. Anemia caused by iron deficiency (iron-deficiency anemia, or IDA) impairs thyroid metabolism function. Both overt and subclinical hypothyroidism are associated with anemia. Previous studies have shown improvement in both conditions with the addition of iron to thyroxine therapy [40]. In conclusion, annual peripheral blood testing with a thyroid panel or minimally TSH (thyroid stimulating hormone), T4 (thyroxine), and T3 levels may be warranted. If peripheral blood chemistry testing reveals abnormalities in calcium or phosphatase, or if a follow-up bone marrow biopsy reveals hypocellular bone marrow, parathyroid testing should be considered.

### 4.2. Predicted Tier 2 Adverse Events Likely Tied to TKI Therapy

As noted in Figure 7, the tier 2 foci comprised approximately the top 5% of predicted nodes relevant to tyrosine kinase. The tier 2 foci were: 6. Inflammation; 7. Kidney function. Inflammation was one of the AEs that occurred frequently in the k-means clustering of CML literature and was especially tied to dasatinib. “Kidney” occurred at the highest frequency in the nilotinib cluster.

#### 4.2.1. Kidney Adverse Events with TKI Therapy

Cluster 16 from the clustering methods, which was dominated by nilotinib, had the term “kidney” occur at a high frequency within the cluster. All text mining analyses revealed a connection between kidney conditions and hematological conditions, namely anemia. SemNet simulations predicted glomerulonephritis, glomerulopathy, toxic nephropathy, and chronic kidney disease as kidney conditions relevant to tyrosine kinase.

Chronic kidney disease (CKD) is characterized by excessive fibroblast activation, and it is hypothesized that tyrosine kinase can increase fibroblast activation and contribute to CKD development. Therefore, it may be valuable to consider the impact of TKIs on fibroblast activation by reversing the effect of tyrosine kinases, and in turn evaluate the impact of TKI treatment on the kidney health of patients. However, most TKIs inhibit more than one kinase, making their exact effect unclear and their individual impact on kidney health difficult to determine [49]. Some studies show imatinib, which inhibits BCR-ABL, c-Kit, and PDGF receptors, to improve the chronic kidney disease of animal models [50]. Nilotinib, which inhibits BCR-ABL and PDGF receptors, improved the thioacetamide-based conditions of animal models [50]. However, other studies showed CML patients with pre-existing renal dysfunctions who took dasatinib and nilotinib experienced an increased risk for acute kidney injury [51]. Another study showed imatinib to decrease renal function in patients with pre-existing renal dysfunction [52]. There are also case reports of dasatinib-induced nephrotic syndrome [53]. Kidney conditions, including chronic kidney insufficiency and general kidney disease, frequently contribute to the development of anemia even in non-CML populations.

Five nodes were identified as relevant to kidney function via analysis of the hub neighborhoods: iron deficiency, PTH, urea, uric acid, and C-reactive protein. While these nodes have clear and heavily weighted ties to the hematological hub nodes, it is unclear whether they are truly important to CML patients on TKI therapy who do not have a history of pre-existing kidney disease. It is possible that the hub node network methodology resulted in the overweighting of the relevance of kidney disease due to its strong connections in each hub’s neighborhood. However, the well-known impact of fluid retention [54] and fluid-related pleural effusion [55] (discussed in Section 4.3.3) amplifies the importance of healthy kidney function for patients with TKIs.

In summary, the results of the present study suggest TKI patients undergo periodic kidney testing, with the periodicity based on the presence of relevant antecedent disease or other risk factors that impact susceptibility. Standard metrics such as serum creatinine, glomerular filtration rate, and blood urea nitrogen are suitable candidates to test for proper kidney function in patients taking TKIs.

#### 4.2.2. Inflammation Adverse Events with TKI Therapy

Inflammation was most represented in cluster 10 (dasatinib) but was also represented in cluster 7 (imatinib) and cluster 16 (nilotinib). “Inflammation” could represent many different types of symptoms. The three highest ranked types of inflammatory disorders in the cross-domain text mining analysis were chronic inflammatory disorder (CID), autoimmune diseases, and periodontitis. Autoimmune disease has been associated with and identified as a predisposing risk factor for getting CML; one study found the odds ratio of getting CML was 1.55 (compared to 1) for patients that had an autoimmune disease prior to being diagnosed with CML [56]. There have also been associations to neurological autoimmune diseases, which are discussed in Section 4.3.2. It was hypothesized that inflammation is an indicator of long-term success in CML. Yet, a more recent study found that biomarkers for inflammation could not predict whether CML patients were more likely to be successful or if they were more likely to relapse when attempting treatment-free remission (TFR), which is a planned cessation of TKI therapy [57]. There have been case reports of colitis followed by dasatinib TKI therapy [58]. Another study has reported that predisposition to pro-inflammation or pro-oxidative stress increases atherothrombotic AEs with TKI therapy, particularly nilotinib [59]. Similarly, dasatinib has been connected to immune-mediated thrombotic thrombocytopenic purpura [60]. Finally, periodontitis was a novel node where there is negligible CML-specific literature mentions. In general, chemotherapy agents are known to increase periodontitis in leukemia [61,62,63], particularly acute myeloid leukemia or acute lymphocytic leukemia (also referred to as acute lymphoblastic leukemia). However, the precise mechanism and strength of association for periodontitis to TKI therapy remains to be seen. In the cross-domain text mining results, periodontitis was the third highest ranked condition in the inflammation foci.

In summary, it appears that inflammation has connections to CML predisposition. Inflammation is most likely a relevant adverse risk factor for patients who have pre-existing inflammatory or autoimmune disorders. Based on the cross-domain text mining predictions of this study and supporting direct literature evidence, it is recommended that inflammatory markers be periodically monitored based on the presence of relevant antecedent disease or other pertinent risk factors that impact susceptibility. Moreover, patients more at risk for clotting should have inflammation markers monitored given inflammation and clotting are known comorbidities even in the general [non-CML] population.

### 4.3. Predicted Tier 3 AEs Likely Tied to TKI Therapy

As noted in Figure 7, the tier 3 foci comprised approximately the top 10% of predicted nodes relevant to tyrosine kinase. The tier 3 foci were: 8. gastrointestinal; 9. neuromuscular; 10. Other. Gastrointestinal events were also frequent in the k-means clustering and accompanied by nearly every TKI. Neuromuscular or neurological symptoms were not as frequent in the k-means clustering. “Other” represented conditions that were in the top 10% of cross-domain text mining relevance predictions but could not be easily categorized into a single overlapping foci.

#### 4.3.1. Gastrointestinal Adverse Events with TKI Therapy

For the cross-domain text mining analysis, bowel regulation (diarrhea or constipation), liver dysfunction, and pancreatitis were among the most highly ranked gastrointestinal events. K-means clustering of CML literature found that “gastrointestinal” was among the most frequent terms and appeared with all TKIs. Gastrointestinal AEs are some of the most common symptoms reported by CML patients. Many patients report nausea, vomiting, and diarrhea. A recent large cross-cohort meta-analysis extensively examined gastrointestinal AEs with TKI therapy [16]. Briefly, results illustrated bosutinib had the most prevalent and severe gastrointestinal AEs, with dasatinib and imatinib being very similar and nilotinib having the least number of reported gastrointestinal adverse events [16]. Diarrhea is most common early in treatment. However, some patients report more constipation with continued therapy, likely due to therapy-induced habituation. In general, the most common gastrointestinal events tend to abate over time. However, attention must be paid to liver and pancreatic function given overt symptoms may not be present until acute, severe complications arise. A recent large-scale clinical meta-analysis reported the prevalence of toxic hepatopathology [64]. Statistically significant increases in toxic hepatopathology were reported for every TKI except dasatinib [64]. Some patients have been switched to dasatinib following imatinib hepatotoxicity [65]. A recent review article discusses the pharmacokinetics of TKI that lead to specific gastrointestinal AEs [66]. Increases in amylase have been seen in some CML patients treated with TKIs [67]. While pancreatitis is a less frequently reported AE even in quantitative clinical meta-analyses [16], the severity of the AE warrants some prudence in monitoring. Current data suggests that ponatinib has the most frequent prevalence of pancreatitis [68].

In summary, the gastrointestinal focus was a tier 3 focus based on cross-domain text mining predictions. Based on the results of this study and supporting clinical evidence, gastrointestinal AEs should be monitored based primarily on the presence of overt symptoms. Basic liver function can be evaluated via a standard peripheral blood chemistry test. The same standard blood chemistry panel was already recommended for regular monitoring to evaluate some of the TKI AEs within the tier 1 foci. If symptoms warrant, a peripheral blood test of amylase can provide more specific evidence of pancreatitis.

#### 4.3.2. Neuromuscular Adverse Events with TKI Therapy

Based on cross-domain text mining analysis, the most highly ranked neuromuscular TKI AEs included autonomia, neuropathy, muscle pain, and cognitive dysfunction. “Neurological disorders” did not show up as a frequent term in the k-means clustering of CML literature. “Musculoskeletal” appeared relatively often even though the term did not make the top terms list shown in Table 1.

There are mixed clinical findings on the impact of pre-existing or antecedent neurological autoimmune diseases versus neurological autoimmune AEs caused by TKI therapy. Autoimmune neurological diseases, such as myasthenia gravis, have a strong enough association with CML that some have suggested that they should be classified as paraneoplastic neurologic syndrome [69]; however, this specific association is to CML and not necessarily TKIs. Nonetheless, new demyelinating AEs with TKI therapy have been reported with dasatinib [70], especially demyelinating peripheral neuropathy. Peripheral neuropathy has also been reported with imatinib [71]. Some patients with this AE who were treated with dasatinib or imatinib successfully transitioned to nilotinib [72]. Another neurological side effect reported with imatinib is severe demyelination resulting in paralysis from transverse myelitis, vision impairment from optic neuritis, or a combination of the two due to neuromyelitis optica [73]; this demyelination tendency could be related to T-cell activation, although the precise mechanism is unknown. However, nilotinib has also been clinically associated with neurological AEs. There have been reports of dystonia and cognitive deficits with nilotinib [74]. There have also been recent reports of neurocognitive deficits with dasatinib [75]. By far the most common neuromuscular adverse event is musculoskeletal pain, which can occur at any point with TKI treatment [76,77] and as part of withdrawal to the TKI during a planned TFR attempt [78].

In summary, based on the predictions of the present study and corroborating clinical evidence, most neurological or neuromuscular AEs would only require assessment when the patient presents with overt symptoms.

#### 4.3.3. Other Adverse Events with TKI Therapy

In the present study, the “other” focus was used to categorize conditions that were found among the top 10% of predicted relevant conditions to tyrosine kinase. Secondary cancers were frequently occurring nodes. Secondary cancers have been associated with CML both before and after CML diagnosis [56,79,80,81]. Essentially every aforementioned cited study found statistically significant increases in secondary malignancy with CML and TKI therapy. This is not unexpected given most cancer therapies are suspected to increase the risk of secondary malignancies, which may be attributed to increased therapy-related immune suppression.

Other foci conditions predicted as relevant were various forms of vitamin deficiency such as vitamins B6, B12, D, C, and of course, iron, which was ranked enough to be its own tier 1 focus. The majority of these vitamins are tied to hematopoiesis, which explains why they are likely present in these tier 3 foci. Beyond ties to hematopoiesis, there are presently no widespread hypotheses as to how or why vitamin deficiency may be associated with TKI therapy. It is possible TKIs may reduce innate absorption.

Edema was another condition ranked in the top 10% in the cross-domain text mining analysis. Edema was also a frequent term in the k-means clustering of CML literature. Imatinib and dasatinib had strong associations with edema [54]. Moreover, edema is noted as a frequent patient-reported side effect that increases the desire to try TFR [82].

Dermatological or skin conditions were another predicted TKI AE type that belonged to the “other” tier 3 foci. Imatinib has been documented to contribute to the development of rash and skin lesions [83], as is seen in cluster 7 in Table 1, where the terms “rash” and “lesion” occur at the highest frequency in the imatinib cluster.

Pleural effusion is perhaps one of the most well-known acute adverse events with TKI therapy [84]. Dasatinib is documented to induce pleural effusion in some CML patients at higher rates than other TKIs [55]. Here, cluster 10 (the dasatinib cluster) ranked highest for the frequency of the terms “pleural”, “effusion”, and “pulmonary”. Interestingly, pleural effusion was not an event predicted in the top 10% for the cross-domain text mining simulations. Pleural effusion is tied to fluid retention and edema, which were predicted to have very relevant connections to TKIs. The presence of general edema is associated with the risk of pleural effusion occurrence.

In summary, most conditions within the “other” foci only require monitoring when overt symptoms are present. Nonetheless, given the higher prevalence of secondary malignancies [56,80,85], patients should be encouraged to be proactive in standard age-appropriate general population prophylactic screenings. Examples include mammograms and pap smears for females; prostate screenings for males; and colonoscopies for age >50 years.

### 4.4. Limitations and Future Work

Cross-domain text mining via natural language processing (NLP) and machine learning enable expanded types of exploratory analyses that would not be otherwise possible. That is, mining across different domains allows comprehensive aggregation of relationships and perspectives. Compared to traditional manual literature reviews of select studies, cross-domain text mining utilizes machine learning approaches, which minimize the interjection of human bias into the results. However, cross-domain text mining’s current major limitation is that nuanced information is difficult to include in automated analysis. For example, the present cross-domain text mining study could not specify certain features for inclusion, such as TKI dose, length of TKI treatment, etc. Future work with patient-intervention-outcome (PIO) NLP models [86] could provide more granularity to optimize the breadth of prediction by knowledge graphs with the detailed features included in traditional clinical cohort meta-analyses or manual systematic reviews. Finally, post-simulation systems like CompositeView [87] could expedite aggregation of cross-domain text mining simulation rankings into foci based on composite scores.

## 5. Conclusions

The need for long-term tyrosine kinase inhibitor (TKI) therapy to treat CML warrants careful patient monitoring for adverse events or side effects. Assessment of CML TKI literature revealed hematological adverse events (anemia, thrombocytopenia, neutropenia) were frequently cited with all TKIs. Clustering revealed significant (*p* < 0.05) differences in AE term frequency by TKI type: gastrointestinal (bosutinib); edema (imatinib); pulmonary (dasatinib); diabetes (nilotinib); cardiovascular (ponatinib). The novel, cross-domain text mining using link prediction and hub node network analysis predicted three tiers of TKI AEs spanning ten physiological foci. Tier 1 foci (top 1% by predicted relevance to tyrosine kinase) included hematological events, glucose, iron, cardiovascular, and thyroid conditions. Tier 2 foci (top 5% by predicted relevance to tyrosine kinase) included inflammation and kidney conditions. Tier 3 foci (top 10% by predicted relevance to tyrosine kinase) include gastrointestinal, neuromuscular or “other” conditions. In summary, while TKIs have been pivotal in changing the outcome of CML, these medications are not benign. Careful proactive monitoring and selection of TKI to minimize patient-specific AEs is necessary to optimize clinical management and patient quality of life.

## Figures and Tables

**Table 1:** Color coding corresponds to colors of clusters in the cluster plot (Figure 4). The terms and clustering frequency illustrate the frequency of different adverse events across each cluster. Maximum frequency across all clusters is indicated with boldfaced text.

Event	C4 Bosutinib	C7 Imatinib	C8 Ponatinib	C10 Dasatinib	C16 Nilotinib
diarrhea	**0.045**	0.005	0.000	0.010	0.001
gastrointestinal	0.017	**0.034**	0.000	0.020	0.006
nausea	**0.016**	0.007	0.000	0.002	0.001
vomiting	**0.014**	0.002	0.000	0.000	0.001
cardiovascular	0.014	0.000	**0.051**	0.004	0.031
pleural	0.011	0.003	0.000	**0.029**	0.002
effusion	0.011	0.005	0.000	**0.032**	0.002
pulmonary	0.009	0.001	0.000	**0.066**	0.002
edema	0.004	**0.020**	0.000	0.001	0.006
rash	0.003	**0.028**	0.002	0.011	0.009
liver	0.008	**0.022**	0.006	0.000	0.013
platelet	0.000	0.019	0.021	**0.028**	0.005
myelosuppression	0.003	**0.012**	0.002	0.007	0.000
hepatitis	0.000	**0.012**	0.000	0.003	0.000
hematological	0.000	**0.011**	0.002	0.004	0.006
hypertension	0.000	0.000	0.019	**0.037**	0.003
thrombocytopenia	0.005	0.007	0.004	0.004	0.001
pneumonia	0.000	0.000	0.000	0.012	0.005
inflammation	0.000	0.007	0.000	**0.012**	0.002
vascular	0.004	0.002	**0.035**	0.022	0.025
arterial	0.000	0.000	0.021	**0.029**	0.024
diabetes	0.000	0.000	0.000	0.000	**0.013**
lesion	0.000	**0.020**	0.002	0.000	0.013

## Data Availability

Access to the SemNet software used to perform cross-domain text mining in this study can be found at https://github.com/pathology-dynamics/ (accessed on 25 July 2022).

## Appendix A

Bag-of-word analysis and k-means clustering was used to assign a label to each cluster as primarily representing a single TKI. The raw cluster frequencies computed to assign labels are shown in Table A1.

**Table A1:** Term frequency of each TKI within each cluster. Color coding corresponds to the colors of clusters in the cluster plot from Figure 4. Maximum frequency across all clusters is indicated with boldfaced text.

TKI/Cluster	4	7	8	10	16
bosutinib	**0.493**	0.003	0.034	0.007	0.004
imatinib	0.053	**0.255**	0.024	0.033	0.078
ponatinib	0.003	0.002	**0.453**	0.000	0.004
dasatinib	0.026	0.009	0.023	**0.367**	0.062
nilotinib	0.027	0.008	0.037	0.011	**0.346**

## References

[1] SEER Chronic Myeloid Leukemia—Cancer Stat Facts. https://seer.cancer.gov/statfacts/html/cmyl.html.

[2] Athale U., Hijiya N., Patterson B.C., Bergsagel J., Andolina J.R., Bittencourt H., Schultz K.R., Burke M.J., Redell M.S., Kolb E.A. (2019). Management of chronic myeloid leukemia in children and adolescents: Recommendations from the Children’s Oncology Group CML Working Group. Pediatr. Blood Cancer.

[3] Massimino M., Stella S., Tirro E., Pennisi M.S., Vitale S.R., Puma A., Romano C., di Gregorio S., Tomarchio C., Di Raimondo F. (2020). ABL1-Directed Inhibitors for CML: Efficacy, Resistance and Future Perspectives. Anticancer Res..

[4] Rea D., Cayuela J.M. (2018). Treatment-free remission in patients with chronic myeloid leukemia. Int. J. Hematol..

[5] Atallah E., Sweet K. (2021). Treatment-Free Remission: The New Goal in CML Therapy. Curr. Hematol. Malig. Rep..

[6] Mahon F.X., Etienne G. (2014). Deep molecular response in chronic myeloid leukemia: The new goal of therapy?. Clin. Cancer Res..

[7] Cortes J., Rea D., Lipton J.H. (2019). Treatment-free remission with first- and second-generation tyrosine kinase inhibitors. Am. J. Hematol..

[8] Rousselot P., Huguet F., Rea D., Legros L., Cayuela J.M., Maarek O., Blanchet O., Marit G., Gluckman E., Reiffers J. (2007). Imatinib mesylate discontinuation in patients with chronic myelogenous leukemia in complete molecular remission for more than 2 years. Blood.

[9] Ross D.M., Branford S., Seymour J.F., Schwarer A.P., Arthur C., Yeung D.T., Dang P., Goyne J.M., Slader C., Filshie R.J. (2013). Safety and efficacy of imatinib cessation for CML patients with stable undetectable minimal residual disease: Results from the TWISTER study. Blood.

[10] Saussele S., Richter J., Guilhot J., Gruber F.X., Hjorth-Hansen H., Almeida A., Janssen J., Mayer J., Koskenvesa P., Panayiotidis P. (2018). Discontinuation of tyrosine kinase inhibitor therapy in chronic myeloid leukaemia (EURO-SKI): A prespecified interim analysis of a prospective, multicentre, non-randomised, trial. Lancet Oncol..

[11] Ross D.M., Masszi T., Gomez Casares M.T., Hellmann A., Stentoft J., Conneally E., Garcia-Gutierrez V., Gattermann N., le Coutre P.D., Martino B. (2018). Durable treatment-free remission in patients with chronic myeloid leukemia in chronic phase following frontline nilotinib: 96-week update of the ENESTfreedom study. J. Cancer Res. Clin. Oncol..

[12] Hughes T.P., Clementino N.C.D., Fominykh M., Lipton J.H., Turkina A.G., Moiraghi E.B., Nicolini F.E., Takahashi N., Sacha T., Kim D.W. (2021). Long-term treatment-free remission in patients with chronic myeloid leukemia after second-line nilotinib: ENESTop 5-year update. Leukemia.

[13] Shah N.P., Garcia-Gutierrez V., Jimenez-Velasco A., Larson S., Saussele S., Rea D., Mahon F.X., Levy M.Y., Gomez-Casares M.T., Pane F. (2020). Dasatinib discontinuation in patients with chronic-phase chronic myeloid leukemia and stable deep molecular response: The DASFREE study. Leuk. Lymphoma.

[14] Shih Y.T., Cortes J.E., Kantarjian H.M. (2019). Treatment value of second-generation BCR-ABL1 tyrosine kinase inhibitors compared with imatinib to achieve treatment-free remission in patients with chronic myeloid leukaemia: A modelling study. Lancet Haematol..

[15] Reff M.J., Shillingburg A., Shah B., Elder C., Prescott H., Kennerly-Shah J. (2020). Front-line use of tyrosine kinase inhibitors in chronic phase chronic myeloid leukemia: Practice considerations. J. Oncol. Pharm. Pract..

[16] Mohanavelu P., Mutnick M., Mehra N., White B., Kudrimoti S., Hernandez Kluesner K., Chen X., Nguyen T., Horlander E., Thenot H. (2021). Meta-Analysis of Gastrointestinal Adverse Events from Tyrosine Kinase Inhibitors for Chronic Myeloid Leukemia. Cancers.

[17] Chan O., Talati C., Isenalumhe L., Shams S., Nodzon L., Fradley M., Sweet K., Pinilla-Ibarz J. (2020). Side-effects profile and outcomes of ponatinib in the treatment of chronic myeloid leukemia. Blood Adv..

[18] Abruzzese E., Mauro M., Apperley J., Chelysheva E. (2020). Tyrosine kinase inhibitors and pregnancy in chronic myeloid leukemia: Opinion, evidence, and recommendations. Ther. Adv. Hematol..

[19] Hughes T., White D. (2013). Which TKI? An embarrassment of riches for chronic myeloid leukemia patients. Hematol. Am. Soc. Hematol. Educ. Program.

[20] Steegmann J.L., Baccarani M., Breccia M., Casado L.F., Garcia-Gutierrez V., Hochhaus A., Kim D.W., Kim T.D., Khoury H.J., Le Coutre P. (2016). European LeukemiaNet recommendations for the management and avoidance of adverse events of treatment in chronic myeloid leukaemia. Leukemia.

[21] Kantarjian H., O’Brien S., Jabbour E., Garcia-Manero G., Quintas-Cardama A., Shan J., Rios M.B., Ravandi F., Faderl S., Kadia T. (2012). Improved survival in chronic myeloid leukemia since the introduction of imatinib therapy: A single-institution historical experience. Blood.

[22] Kirkpatrick A.O.C., Kartchner D., Allegri S., Nakajima An D., McCoy K., Davalbhakta E., Mitchell C.S. (2022). Optimizations for Computing Relatedness in Biomedical Heterogeneous Information Networks: SemNet 2.0. Big Data Cogn. Comput..

[23] Sedler A.R., Mitchell C.S. (2019). SemNet: Using Local Features to Navigate the Biomedical Concept Graph. Front. Bioeng. Biotechnol..

[24] Xiao Y., Zhang B., Cloyd J.M., Alaimo L., Xu G., Du S., Mao Y., Pawlik T.M. (2022). Novel Drug Candidate Prediction for Intrahepatic Cholangiocarcinoma via Hub Gene Network Analysis and Connectivity Mapping. Cancers.

[25] Wu Z.W., Gao Z.R., Liang H., Fang T., Wang Y., Du Z.Q., Yang C.X. (2022). Network analysis reveals different hub genes and molecular pathways for pig in vitro fertilized early embryos and parthenogenotes. Reprod. Domest. Anim..

[26] Nemati M., Zare N., Hedayat-Evrigh N., Asghari R. (2022). Identification of Key Gene Network Modules and Hub Genes Associated with Wheat Response to Biotic Stress Using Combined Microarray Meta-analysis and WGCN Analysis. Mol. Biotechnol..

[27] Zhang Y., Jin R., Zhou Z.-H. (2010). Understanding bag-of-words model: A statistical framework. Int. J. Mach. Learn. Cybern..

[28] MacQueen J. Some methods for classification and analysis of multivariate observations. Proceedings of the Fifth Berkeley Symposium on Mathematical Statistics and Probability.

[29] Pedregosa F.V.G., Gramfort A., Michel V., Thirion B., Grisel O., Blondel M., Prettenhofer P., Weiss R., Dubourg V., Vanderplas J. (2011). Scikit-learn: Machine Learning in Python. J. Mach. Learn. Res..

[30] McCoy K., Gudapati S., He L., Horlander E., Kartchner D., Kulkarni S., Mehra N., Prakash J., Thenot H., Vanga S.V. (2021). Biomedical Text Link Prediction for Drug Discovery: A Case Study with COVID-19. Pharmaceutics.

[31] Huang Z.A., Huang Y.A., You Z.H., Zhu Z., Sun Y. (2018). Novel link prediction for large-scale miRNA-lncRNA interaction network in a bipartite graph. BMC Med. Genom..

[32] Guven C., Atzmueller M. (2019). Applying Answer Set Programming for Knowledge-Based Link Prediction on Social Interaction Networks. Front. Big Data.

[33] Crichton G., Guo Y., Pyysalo S., Korhonen A. (2018). Neural networks for link prediction in realistic biomedical graphs: A multi-dimensional evaluation of graph embedding-based approaches. BMC Bioinform..

[34] Gitelman S.E., Bundy B.N., Ferrannini E., Lim N., Blanchfield J.L., DiMeglio L.A., Felner E.I., Gaglia J.L., Gottlieb P.A., Long S.A. (2021). Imatinib therapy for patients with recent-onset type 1 diabetes: A multicentre, randomised, double-blind, placebo-controlled, phase 2 trial. Lancet Diabetes Endocrinol..

[35] Fountas A., Diamantopoulos L.N., Tsatsoulis A. (2015). Tyrosine Kinase Inhibitors and Diabetes: A Novel Treatment Paradigm?. Trends Endocrinol. Metab..

[36] Elsherbiny N.M., El-Sherbiny M., Said E. (2015). Amelioration of experimentally induced diabetic nephropathy and renal damage by nilotinib. J. Physiol. Biochem..

[37] Samaha M.M., Said E., Salem H.A. (2019). Nilotinib enhances beta-islets integrity and secretory functions in a rat model of STZ-induced diabetes mellitus. Eur. J. Pharmacol..

[38] Valent P., Gastl G., Geissler K., Greil R., Hantschel O., Lang A., Linkesch W., Lion T., Petzer A.L., Pittermann E. (2012). Nilotinib as frontline and second-line therapy in chronic myeloid leukemia: Open questions. Crit. Rev. Oncol. Hematol..

[39] Roden M. (2016). Diabetes mellitus: Definition, classification and diagnosis. Wien. Klin. Wochenschr..

[40] Soliman A.T., De Sanctis V., Yassin M., Wagdy M., Soliman N. (2017). Chronic anemia and thyroid function. Acta Bio-Med. Atenei Parm..

[41] Kim D.S., Na Y.J., Kang M.H., Yoon S.Y., Choi C.W. (2016). Use of deferasirox, an iron chelator, to overcome imatinib resistance of chronic myeloid leukemia cells. Korean J. Intern Med..

[42] Medeiros B.C., Possick J., Fradley M. (2018). Cardiovascular, pulmonary, and metabolic toxicities complicating tyrosine kinase inhibitor therapy in chronic myeloid leukemia: Strategies for monitoring, detecting, and managing. Blood Rev..

[43] Jain P., Kantarjian H., Boddu P.C., Nogueras-Gonzalez G.M., Verstovsek S., Garcia-Manero G., Borthakur G., Sasaki K., Kadia T.M., Sam P. (2019). Analysis of cardiovascular and arteriothrombotic adverse events in chronic-phase CML patients after frontline TKIs. Blood Adv..

[44] Mauro M.J. (2021). Lifelong TKI therapy: How to manage cardiovascular and other risks. Hematol. Am. Soc. Hematol. Educ. Program.

[45] Ahmadieh H., Salti I. (2013). Tyrosine kinase inhibitors induced thyroid dysfunction: A review of its incidence, pathophysiology, clinical relevance, and treatment. BioMed Res. Int..

[46] Patel S., Nayernama A., Jones S.C., de Claro R.A., Waldron P.E. (2020). BCR-ABL1 tyrosine kinase inhibitor-associated thyroid dysfunction: A review of cases reported to the FDA Adverse Event Reporting System and published in the literature. Am. J. Hematol..

[47] Lim D.J., Oh E.J., Park C.W., Kwon H.S., Hong E.J., Yoon K.H., Kang M.I., Cha B.Y., Lee K.W., Son H.Y. (2007). Pancytopenia and secondary myelofibrosis could be induced by primary hyperparathyroidism. Int. J. Lab. Hematol..

[48] O’Sullivan S., Lin J.M., Watson M., Callon K., Tong P.C., Naot D., Horne A., Aati O., Porteous F., Gamble G. (2011). The skeletal effects of the tyrosine kinase inhibitor nilotinib. Bone.

[49] Wang J., Zhuang S. (2017). Src family kinases in chronic kidney disease. Am. J. Physiol. Ren. Physiol..

[50] Iyoda M., Shibata T., Hirai Y., Kuno Y., Akizawa T. (2011). Nilotinib attenuates renal injury and prolongs survival in chronic kidney disease. J. Am. Soc. Nephrol..

[51] Sasaki K., Lahoti A., Jabbour E., Jain P., Pierce S., Borthakur G., Daver N., Kadia T., Pemmaraju N., Ferrajoli A. (2016). Clinical Safety and Efficacy of Nilotinib or Dasatinib in Patients with Newly Diagnosed Chronic-Phase Chronic Myelogenous Leukemia and Pre-Existing Liver and/or Renal Dysfunction. Clin. Lymphoma Myeloma Leuk..

[52] Tong W.G., Kantarjian H., O’Brien S., Faderl S., Ravandi F., Borthakur G., Shan J., Pierce S., Rios M.B., Cortes J. (2010). Imatinib front-line therapy is safe and effective in patients with chronic myelogenous leukemia with pre-existing liver and/or renal dysfunction. Cancer.

[53] ElShaer A., Almasry M., Alawar M., Masoud H., El Kinge A.R. (2021). Dasatinib-Induced Nephrotic Syndrome: A Case Report. Cureus.

[54] Masiello D., Gorospe G., Yang A.S. (2009). The occurrence and management of fluid retention associated with TKI therapy in CML, with a focus on dasatinib. J. Hematol. Oncol..

[55] Hailan Y.M., Elyas A., Abdulla M.A., Yassin M.A. (2021). Dasatinib-Induced Pleural and Pericardial Effusions. Cureus.

[56] Gunnarsson N., Hoglund M., Stenke L., Wallberg-Jonsson S., Sandin F., Bjorkholm M., Dreimane A., Lambe M., Markevarn B., Olsson-Stromberg U. (2016). Increased prevalence of prior malignancies and autoimmune diseases in patients diagnosed with chronic myeloid leukemia. Leukemia.

[57] Soderlund S., Persson I., Ilander M., Guilhot J., Hjorth-Hansen H., Koskenvesa P., Richter J., Saussele S., Mustjoki S., Olsson-Stromberg U. (2020). Plasma proteomics of biomarkers for inflammation or cancer cannot predict relapse in chronic myeloid leukaemia patients stopping tyrosine kinase inhibitor therapy. Leuk. Res..

[58] Oshima N., Mishima Y., Shibagaki K., Kawashima K., Ishimura N., Ikejiri F., Onishi C., Okada T., Inoue M., Moriyama I. (2021). Differential gene expression analysis of dasatinib-induced colitis in a patient with chronic myeloid leukemia followed for 3 years: A case report. BMC Gastroenterol..

[59] Bocchia M., Galimberti S., Aprile L., Sicuranza A., Gozzini A., Santilli F., Abruzzese E., Barate C., Scappini B., Fontanelli G. (2016). Genetic predisposition and induced pro-inflammatory/pro-oxidative status may play a role in increased atherothrombotic events in nilotinib treated chronic myeloid leukemia patients. Oncotarget.

[60] Demirsoy E.T., Mehtap O., Atesoglu E.B., Tarkun P., Eren N., Geduk A., Hacihanefioglu A. (2018). Dasatinib-induced immune mediated-thrombotic thrombocytopenic purpura. Transfus. Apher. Sci..

[61] Ptasiewicz M., Maksymiuk P., Chalas R. (2022). Oral Hygiene Considerations in Adult Patients with Leukemia during a Cycle of Chemotherapy. Int. J. Environ. Res. Public Health.

[62] Ashok L., Sujatha G.P., Hema G. (2010). Estimation of salivary amylase and total proteins in leukemia patients and its correlation with clinical feature and radiographic finding. Indian J. Dent. Res..

[63] Allareddy V., Prakasam S., Allareddy V., Martinez-Schlurmann N.I., Rampa S., Nalliah R.P., Eswaran S.V., Elangovan S. (2015). Poor Oral Health Linked with Increased Risk of Infectious Complications in Adults with Leukemia. J. Mass. Dent. Soc..

[64] Wang Z., Wang X., Wang Z., Feng Y., Jia Y., Jiang L., Xia Y., Cao J., Liu Y. (2021). Comparison of Hepatotoxicity Associated With New BCR-ABL Tyrosine Kinase Inhibitors vs Imatinib Among Patients with Chronic Myeloid Leukemia: A Systematic Review and Meta-analysis. JAMA Netw. Open.

[65] Harbaum L., Marx A., Goekkurt E., Schafhausen P., Atanackovic D. (2014). Treatment with dasatinib for chronic myeloid leukemia following imatinib-induced hepatotoxicity. Int. J. Hematol..

[66] Kumar V., Singh P., Gupta S.K., Ali V., Verma M. (2022). Transport and metabolism of tyrosine kinase inhibitors associated with chronic myeloid leukemia therapy: A review. Mol. Cell Biochem..

[67] Nesr G., Claudiani S., Khorashad J., Apperley J., Milojkovic D. (2019). The influence of salivary amylase on total amylase elevation in CML patients treated with TKI therapy: A case series of 3 patients. Leuk. Lymphoma.

[68] Shamroe C.L., Comeau J.M. (2013). Ponatinib: A new tyrosine kinase inhibitor for the treatment of chronic myeloid leukemia and Philadelphia chromosome-positive acute lymphoblastic leukemia. Ann. Pharmacother..

[69] Sasi S., Mohamed M., Chitrambika P., Yassin M. (2022). Myasthenia Gravis and Myeloproliferative Neoplasms—Mere Association or Paraneoplastic Neurologic Syndrome: A Mini-Review. Acta Bio-Med. Atenei Parm..

[70] Ishida T., Akagawa N., Miyata T., Tominaga N., Iizuka T., Higashihara M., Suzuki T., Miyazaki K. (2018). Dasatinib-associated reversible demyelinating peripheral polyneuropathy in a case of chronic myeloid leukemia. Int. J. Hematol..

[71] Kavanagh S., Bril V., Lipton J.H. (2018). Peripheral neuropathy associated with imatinib therapy for chronic myeloid leukemia. Blood Res..

[72] Inoue H., Taji H., Yamada K., Iriyama C., Saito T., Kato H., Yanada M., Yamamoto K., Matsukawa N. (2020). Dasatinib-induced Reversible Demyelinating Peripheral Neuropathy and Successful Conversion to Nilotinib in Chronic Myelogenous Leukemia. Intern. Med..

[73] Rotstein D.L., Sawicka K., Bharatha A., Montalban X., Lipton J.H. (2020). CNS demyelination after initiating the tyrosine kinase inhibitor imatinib: A report of two cases. Mult. Scler..

[74] Chan J., Shah P., Moguel-Cobos G. (2019). Nilotinib-Induced Dystonia and Cognitive Deficits in a Neurologically Normal Patient with Chronic Myeloid Leukemia. Case Rep. Neurol. Med..

[75] Chamoun K., Rabinovich E., Baer L., Fastenau P., Lima M. (2020). A case of neurocognitive deficit strongly related to dasatinib therapy. Hematol. Transfus. Cell Ther..

[76] Chow E.J., Doody D.R., Wilkes J.J., Becker L.K., Chennupati S., Morin P.E., Winestone L.E., Henk H.J., Lyman G.H. (2021). Adverse events among chronic myelogenous leukemia patients treated with tyrosine kinase inhibitors: A real-world analysis of health plan enrollees. Leuk. Lymphoma.

[77] Yu L., Huang X., Gale R.P., Wang H., Jiang Q. (2019). Variables associated with patient-reported symptoms in persons with chronic phase chronic myeloid leukemia receiving tyrosine kinase inhibitor therapy. Medicine.

[78] Breccia M., Efficace F., Colafigli G., Scalzulli E., Di Prima A., Martelli M., Foa R. (2020). Tyrosine kinase inhibitor discontinuation in the management of chronic myeloid leukemia: A critical review of the current practice. Expert Rev. Hematol..

[79] Duman B.B., Paydas S., Disel U., Besen A., Gurkan E. (2012). Secondary malignancy after imatinib therapy: Eight cases and review of the literature. Leuk. Lymphoma.

[80] Kumar V., Garg M., Chaudhary N., Chandra A.B. (2018). An observational study on risk of secondary cancers in chronic myeloid leukemia patients in the TKI era in the United States. PeerJ.

[81] Miranda M.B., Lauseker M., Kraus M.P., Proetel U., Hanfstein B., Fabarius A., Baerlocher G.M., Heim D., Hossfeld D.K., Kolb H.J. (2016). Secondary malignancies in chronic myeloid leukemia patients after imatinib-based treatment: Long-term observation in CML Study IV. Leukemia.

[82] Narra R.K., Flynn K.E., Atallah E. (2017). Chronic Myeloid Leukemia-the Promise of Tyrosine Kinase Inhibitor Discontinuation. Curr Hematol. Malig. Rep..

[83] Albayrak M., Celebi H., Albayrak A., Can E.S., Aslan V., Onec B., Coban I. (2011). Serious skin reaction associated with imatinib in a patient with chronic myeloid leukemia. Eurasian J. Med..

[84] Cortes J.E., Jimenez C.A., Mauro M.J., Geyer A., Pinilla-Ibarz J., Smith B.D. (2017). Pleural Effusion in Dasatinib-Treated Patients with Chronic Myeloid Leukemia in Chronic Phase: Identification and Management. Clin. Lymphoma Myeloma Leuk..

[85] Gunnarsson N., Stenke L., Hoglund M., Sandin F., Bjorkholm M., Dreimane A., Lambe M., Markevarn B., Olsson-Stromberg U., Richter J. (2015). Second malignancies following treatment of chronic myeloid leukaemia in the tyrosine kinase inhibitor era. Br. J. Haematol..

[86] Nye B., Jessy Li J., Patel R., Yang Y., Marshall I.J., Nenkova A., Wallace B.C. (2018). A Corpus with Multi-Level Annotations of Patients, Interventions and Outcomes to Support Language Processing for Medical Literature. Proc. Conf. Assoc. Comput. Linguist Meet..

[87] Allegri S.A., McCoy K., Mitchell C.S. (2022). CompositeView: A Network-Based Visualization Tool. Big Data Cogn. Comput..

